# Sex Differences in Renal Cell Carcinoma: The Importance of Body Composition

**DOI:** 10.1245/s10434-022-12738-z

**Published:** 2022-11-09

**Authors:** Sebastian Dahlmann, Keno Bressem, Behschad Bashian, Sevtap Tugce Ulas, Maximilian Rattunde, Felix Busch, Marcus R. Makowski, Katharina Ziegeler, Lisa Adams

**Affiliations:** 1 Department of Radiology, Charité, Berlin, Germany; 2 Berlin Institute of Health at Charité – Universitätsmedizin Berlin, Berlin, Germany; 3 Department of Radiology, Technical University of Munich, Munich, Germany; 4 Department of Radiology, Stanford University, Stanford, CA USA

## Abstract

**Purpose:**

To examine sex-specific differences in renal cell carcinoma (RCC) in relation to abdominal fat accumulation, psoas muscle density, tumor size, pathology, and survival, and to evaluate possible associations with RCC characteristics and outcome.

**Methods:**

A total of 470 patients with RCC who underwent nephrectomy between 2006 and 2019 were included in this retrospective study. Specific characteristics of RCC patients were collected, including sex, height, tumor size, grade, and data on patient survival, if available. Abdominal fat measurements and psoas muscle area were determined at the level of L3 (cm^2^).

**Results:**

Women had a higher subcutaneous (*p* < 0.001) and men had a higher visceral fat area, relative proportion of visceral fat area (*p* < 0.001), and psoas muscle index (*p* < 0.001). Logistic regression analysis showed an association between higher psoas muscle index and lower grade tumors [women: odds ratio (OR) 0.94, 95% confidence interval (CI) 0.89–0.99, *p* = 0.011; men: OR 0.97 (95% CI, 0.95–0.99, *p* = 0.012]. Univariate regression analysis demonstrated an association between psoas muscle index and overall survival (women: OR 1.41, 95% CI 1.03–1.93, *p* = 0.033; men: OR 1.62 (95% CI, 1.33–1.97, *p* < 0.001). In contrast, there were no associations between abdominal fat measurements and tumor size, grade, or survival. Also, there were no sex-specific differences in tumor size or tumor grades.

**Conclusions:**

A higher preoperative psoas muscle index was independently associated with overall survival in RCC patients, with a stronger association in men compared with women. In addition, the psoas muscle index showed an inverse association with tumor grade, whereby this association was slightly more pronounced in women than in men.

Sex differences in renal cell carcinoma (RCC) may be due to a combination of genetic, lifestyle, environmental, and epigenetic factors.^1^ The risk of developing RCC appears to be twice as high in men as in women, with larger and higher grade tumors more often occurring in men.^2^ Apart from genetic differences^3^, lifestyle factors and associated risks (such as smoking, diet, physical activity) also play an important role in the development of RCC and influence disease progression or therapy response. Some risk factors, such as smoking (causes 22.4% of RCC in men and 8.4% in women) and hypertension (1.34 higher risk of developing RCC in men), are more prevalent in men than in women.^4^ Other risk factors, such as obesity (2.5-fold increase in risk for RCC) seem to affect women and men equally.^5^ However, the association between abdominal obesity and RCC is often stronger in men, although a positive association has also been observed in women.^6^ One possible explanation could be differences in body composition in terms of fat distribution and muscle mass. While men often store more visceral fat and have more muscle mass on average, women—varying by hormone levels and age—tend to store more subcutaneous fat and have proportionally less muscle.^7^ Unlike subcutaneous fat, visceral fat is known to contribute to metabolic syndrome and increased cardiovascular risk.^8^ Regarding sarcopenia, there is also evidence of a negative impact on mortality and disease recurrence in RCC patients.^9,10^

While research on body composition and sex and gender-sensitive medicine is on the rise, holistic gender-specific analyses focusing on both abdominal fat distribution and muscle density in relation to tumor size, grade, survival, and pathology in RCC are currently lacking.

Therefore, the aims of this study were to: (1) evaluate sex-specific differences in abdominal fat and psoas muscle measurements in RCC patients; and (2) determine sex-specific differences in RCC in terms of male/female ratio, tumor size, histology (grade, type), and overall survival. The overall objective was to simultaneously investigate sex-specific differences in RCC in terms of abdominal fat accumulation, psoas muscle density, tumor size, and pathology, and to evaluate possible associations with RCC characteristics.

## Materials and Methods

### Study Population

In total, 470 patients were included in the present retrospective analysis. CT data of patients with RCC who received CT imaging between January 2006 and October 2019 at Charité University hospital were analyzed. Inclusion criteria were contrast-enhanced preoperative CT imaging with histologic confirmation of RCC within 1 year of CT imaging. Exclusion of patients occurred when the CT examination and histology were more than a year apart.

This retrospective study was approved by the Institutional Review Board, including a waiver of informed consent (EA1/201/17). It was conducted in accordance with the local regulations, following the Declaration of Helsinki as well as Good Clinical Practice.

### CT Imaging

The CT scanners used for imaging were a Canon Aquilion 64 (64 slice), an Aquilion Prime (80 slice), an Aquilion One (320 slice) (Canon Medical Systems, Ōtawara, Japan), and a GE Lightspeed VCT (GEL) scanner. Iodine-containing contrast agents such as Accupaque 350 (GE Healthcare, Chicago, Ill, USA) and Ultravist 370 (Bayer Vital GmbH, Leverkusen, Germany) were used in weight-adapted doses. The standard examination algorithm consisted of an initial arterial examination of the upper abdomen 40 s after the start of the machine contrast injection and a subsequent venous phase of the abdomen and pelvis 120 s after contrast injection.

### Evaluation and Segmentation of Images

Based on preoperative CT imaging and patient records, specific characteristics of the RCC patients were recorded. This included the sex of the patients, their height, the maximum transverse diameter of the tumor, as well as the tumor volume in cm^3^, tumor entity, grading, and data on patient survival, if available.

For tumor volume segmentation, CT examinations in the Digital Imaging and Communications in Medicine format were anonymized and transferred to a local workstation. ITK-Snap (Version 3.8.0) was used for multi-slice segmentation of the tumor region (enabling subsequent 3D tumor volume assessment).

For fat quantification in CT, we used Vital’s Vitrea™ Advanced Visualization postprocessing applications (Version 7.0, Canon Medical Systems Corporation, Ōtawara, Tochigi, Japan). This enabled automated quantification of subcutaneous and visceral fat tissue based on a single slice of non-contrast-enhanced CT data at the L3 lumbar vertebra landmark. In the same slice, the psoas muscle area was determined by using a freehand region of interest (ROI) method. As a result, the visceral fat area (VFA), subcutaneous fat area (SFA), relative visceral fat area (rVFA), total fat area (TFA) and psoas muscle area (PMA) could be quantified. Based on the PMA and the patient’s height, the psoas muscle index (PMI) was calculated as follows: (PMA_right_ + PMA_left_(cm^2^))/height(m^2^).^11^ The psoas muscle index, particularly at the level L3, is an easy-to-acquire imaging marker of sarcopenia and was previously shown to be representative of the muscle volume.^12^

The collected data were saved in tabular form and then exported as comma-separated values for subsequent statistical analysis.

### Statistical Analysis

Statistical analysis was performed using *R* (version 4.1.2) and the packages “tidyverse” (version 1.3.1.) and “survival” (version 3.2-13). The Shapiro–Wilk test was used to test continuous variables for normal distribution. The median and interquartile range (IQR) were provided in case of non-normal distribution. The Kruskal–Wallis test or Wilcoxon rank-sum test were used to compare the distribution of non-parametric RCC characteristics (e.g., grade and tumor stage) between men and women. In addition, analysis of variance (ANOVA), as well as uni- and multivariate regression analyses were conducted. Box plots were created to display sex-specific differences for count data. Kaplan-Meier curves were created to display survival over time. A Cox proportional hazards model was used to assess the association between several variables and survival. A *p* value <0.05 was considered statistically significant.

## Results

### Study Population Characteristics

**Fig. 1:**
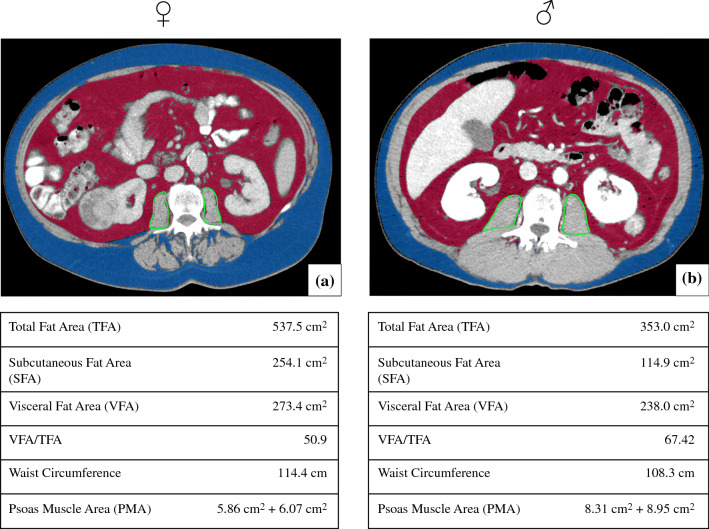
An example fat and muscle segmentation in female and male patients. Segmentation of the fat tissue was automatic, while the psoas muscle region was segmented using a freehand region of interest (ROI)

The study population consisted of 470 patients with histologically confirmed RCC (319 men, 151 women) who ranged in age from 27 to 90 years (median, interquartile range, 64 years, 16 years). Patient clinical and pathologic characteristics stratified by sex are provided in Table 1. Please refer to Fig. 1 for representative examples of CT images used for analysis.

**Table 1:** RCC characteristics and fat measures for the whole population, men, and women, with corresponding *p* values for sex-specific differences

		Total	Men	Women	*P* value
Age	Median, IQR	64, 16	63, 16	65, 16	*p* = 0.003*
Clear-cell RCC	*n*/*n*, %	395/470, 84%	270/322, 84%	125/148, 84%	*p* = 0.87
Papillary RCC	*n*/*n*, %	60/470, 13%	45/322, 14%	15/148, 10%	*p* = 0.25
Chromophobe RCC	*n*/*n*, %	15/470, 3%	7/322, 2%	8/148, 5%	*p* = 0.06
Tumor diameter (cm)	Median, IQR	50, 50	48.5, 48.8	53, 53	*p* = 0.42
Tumor size (cm^3^)	Median, IQR	65.5, 241	62.5, 207.8	88.5, 282.5	*p* = 0.21
Total fat area (cm^2^)	Median, IQR	324.3, 226.4	326.6, 229.1	320.1, 202.2	*p* = 0.72
Subcutaneous fat area (cm^2^)	Median, IQR	151.9, 114	137, 93.2	205.3, 140.4	*p* < 0.001*
Visceral fat area (cm^2^)	Median, IQR	134.7, 147.4	166.2, 161.2	104.3, 100.2	*p* < 0.001*
Relative proportion of visceral fat	Median, IQR	0.5, 0.2	0.5, 0.2	0.3 ± 0.1	*p* < 0.001*
Waist circumference (cm)	Median, IQR	104.3, 16.5	105.8, 14.8	100, 19.5	*p* = 0.001*
Psoas muscle index (cm^2^/m^2^)	Median, IQR	5.4, 2.7	5.9, 2.1	4.8 ± 1.5	*p* < 0.001*
Psoas muscle area (cm^2^)	Median, IQR	14.8, 8.0	16.7, 8.9	11.9, 3.8	*p* < 0.001*

The symbol * indicates statistical significance *p* < 0.05

Routine patient records in Germany do not include patient’s ethnicity and race. As a consequence, these data are not available for this retrospective patient cohort

### Sex Differences in RCC Characteristics and Body Composition Parameters

In line with previous research, RCC was about twice as common in men as in women, with women being slightly older at surgery compared with men (*p* = 0.003). There were no significant differences in tumor size or volume and no significant differences in the distribution of tumor grades (*p* > 0.05). Even though women appeared to have a larger proportion of chromophobe renal cell carcinoma (RCC, 5% versus 3%), this difference was just not significant (*p* = 0.06). Tumor size was related to tumor grade in both sexes (men: *p* = 0.04, women: *p* < 0.001).

**Fig. 2:**
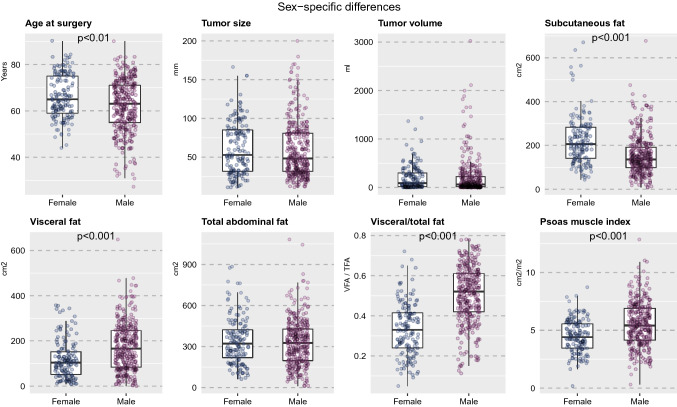
Box plots for illustration of sex-specific differences in age at surgery, tumor volume, subcutaneous fat, visceral fat, total abdominal fat, relative proportion of visceral subcutaneous fat, and psoas muscle index

There were significant sex-specific differences in body composition measurements. While the total fat area was similar between sexes, women had a significantly higher subcutaneous fat area (*p* < 0.001). On the other hand, men had a higher visceral fat area and relative proportion of visceral fat area (*p* < 0.001), waist circumference (*p* = 0.001), and psoas muscle index (*p* < 0.001). Figure 2 provides box plots to illustrate the sex-specific differences in RCC characteristics and body composition parameters. Please refer to Table 1 for an overview of patient characteristics stratified by sex.

### Associations Between Body Composition Parameters, Tumor Size, and Grade

Looking at any potential associations between body composition measurements and tumor grade (low versus high grade), there was a significant association between the psoas muscle index and tumor grade for both women (*p* = 0.012) and men (*p* = 0.017). It could be observed that a higher psoas muscle index was associated with lower grade tumors (5 ± 1.4 vs. 4.3 ± 1.6 in higher grade tumors in women and 6.1 ± 2.1 vs. 5.5 ± 1.8 in higher grade tumors in men). In logistic regression analysis, the odds ratio (OR) for a higher psoas muscle index was 0.94 (95% CI, 0.89–0.99, *p* = 0.011) for women and 0.97 (95% CI, 0.95–0.99, *p* = 0.012) for men, indicating a small, but significant effect on tumor grade in RCC, which was stronger in women than in men. There was no correlation of the psoas muscle index with tumor volume (*p* = 0.10).

Regarding abdominal body fat measurements, there were no significant associations with tumor grade or tumor volume (tumor grade: TFA: *p* = 0.10, VFA: *p* = 0.26, rVFA = 0.11, waist circumference: *p* = 0.10; tumor volume: TFA: *p* = 0.17, VFA: *p* = 0.08, rVFA = 0.09, waist circumference: *p* = 0.49).

### Associations Between Tumor Size and Grade, Body Composition Parameters, and Survival

Survival data were available for 298 out of 470 patients. Of these 298 patients, 50 (35 men and 15 women) died within 10 years and 42 died within 5 years (31 men, 11 women). The 5-year overall survival rate was 85.9%. However, the comparatively high number of missing values (*n* = 172) must be taken into account in the interpretation.

**Fig. 3:**
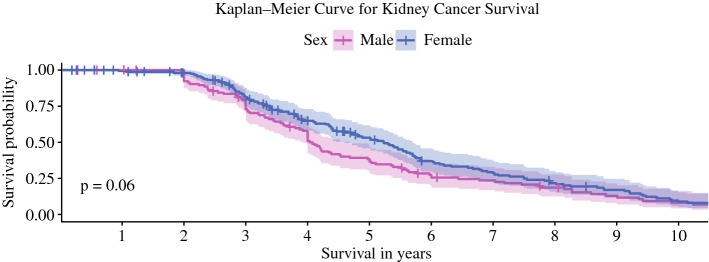
Kaplan-Meier survival curves for men and women who underwent (partial) nephrectomy for kidney cancer. No sex-specific difference in overall survival can be observed

Although there was a trend for slightly higher survival among women, this difference between men and women did not reach statistical significance (*p* = 0.06). Please also refer to Fig. 3 for a Kaplan-Meier survival plot. In the present study, the association between age and survival was also (just) not significant (*p* = 0.07). In addition, no significant association could be found between tumor grade and survival (*p* = 0.35).

**Fig. 4:**
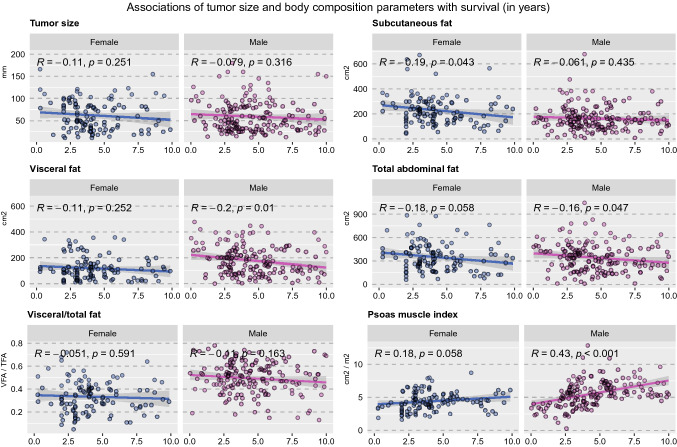
An overview of sex-specific differences in survival from kidney cancer regarding tumor size as well as the body composition markers, which include subcutaneous fat, visceral fat, total abdominal fat, relative proportion of visceral fat, and psoas muscle index

Correlation analysis revealed very weak correlations between total abdominal fat, subcutaneous abdominal fat, visceral fat, and survival (Fig. 4). There were differences between men and women. For women, a weak inverse correlation between subcutaneous fat and survival could be observed (*R* = −0.19, *p* = 0.043). By contrast, men showed weak inverse correlations between visceral fat (*R* = −0.2, *p* = 0.01) and total abdominal fat (*R* = −0.16, *p* = 0.047) and survival. The psoas muscle index showed a weak to moderate positive correlation with survival time for men (*R* = 0.43, *p* < 0.001) and a weak positive correlation with survival time for women (*R* = 0.18, *p* = 0.058).

In multivariate analysis, only the positive association between the psoas muscle index and survival remained significant, with an OR of 1.62 for men (95% CI, 1.33–1.97, *p* < 0.001) and an OR of 1.41 for women (95% CI, 1.03–1.93, *p* = 0.033). Therefore, a higher psoas muscle index was associated with a higher chance of survival, whereby this effect appeared to be stronger in men than in women.

## Discussion

We investigated sex-specific differences in RCC in terms of tumor size, pathology, grade, abdominal fat accumulation, and PMI as an indicator of sarcopenia, with the aim of identifying possible associations with RCC characteristics at the time of diagnosis. As expected, we could demonstrate that RCC was twice as frequent in males as in females. In addition, there were no sex-specific differences in tumor size or grade. While there were differences in abdominal fat measurements between men and women, they were not significantly associated with tumor size, grade, or survival. The PMI, on the other hand, showed a weak inverse association with higher grade tumors, which was slightly more pronounced in women than in men. Furthermore, the PMI showed a weak to moderate positive correlation with survival time in men and women. Multivariate regression analysis confirmed that a higher PMI increased chances of survival, whereby this effect appeared to be stronger in men than in women.

Because sex hormones influence patterns of adipose tissue distribution and body composition, it is important to consider sex-specific differences when investigating the relationship between body composition and RCC characteristics, e.g., size and pathology. CT provides an unparalleled opportunity to precisely quantify tissue distribution and thus body composition, which was confirmed in the International Diabetes Federation consensus statement.^13^ Similarly, CT-based measurements of the psoas muscle have recently been recognized as valid and easy-to-acquire imaging biomarkers for sarcopenia.^14^

So far, several studies have indicated a significant association between visceral adipose tissue and RCC prognosis, survival, subtype, and grade.^15–22^ Regarding sex-specific differences, a previous study reported an association between sex-specific visceral fat composition and high-grade RCC in females.^7^

For RCC outcomes, there are currently several prognostic classification systems, including the PADUA, RENAL, UISS, SSIGN, and MSKCC scores.^23^ For example, the SSIGN score can be used to predict progression of clear cell renal cell carcinoma after radical nephrectomy. So far, several studies have been published on the association between different RCC classification systems and surgical strategies and complications. However, unexplained variability in these prognostic models has driven the search for new prognostic factors, including sex-specific differences and body composition.^24–27^ Hence, it is of clinical interest to investigate abdominal adipose fat tissue and skeletal muscle density in combination because especially high visceral adipose tissue and low muscle density may show an adverse influence on RCC pathology and survival. While the protective effect of a higher PMI could be confirmed in the present study, surprisingly, there were no significant effects of abdominal fat on tumor size, grade, or individual survival. The PMI, however, was positively associated with lower grade tumors and survival.

Sarcopenia is characterized by an age-related reduction in muscle mass that results in decreased muscle size and function.^28^ Clinical manifestation of sarcopenia, also referred to as frailty syndrome, is associated with muscle loss and results in a state of immunosenescence and high vulnerability for adverse health outcomes.^29, 30^ Several previous studies demonstrated that frailty was associated with generally poor postoperative outcomes and higher mortality in older patients.^31,32^ Many current approaches for assessment of frailty rely on combining several health data, e.g., assessment of weakness, unintentional weight loss, exhaustion, and low physical activity.^29^ Given that such an assessment can be time-consuming and is often not available in clinical practice, fast and easy-to-measure imaging markers appear advantageous.

Here, CT-based measurements enable an effective and standardizable measurement of core muscle quality and therefore show potential as ‘one-stop-shop’ imaging biomarkers for sarcopenia. The advantage of the PMI as a marker of sarcopenia is that it is easy to acquire on CT scans and only needs patient height as additional information.

A number of previous studies show that CT-derived psoas muscle measurements, such as PMI, are increasingly used to predict patient outcomes and mortality for different diseases.^33–36^ With regard to RCC, prior research showed an association of sarcopenia with worse prognosis, whereby a low skeletal muscle index was associated with poorer survival.^9, 37,38^ Fukushima et al. defined sarcopenia based on CT measurements of muscle index, and identified sarcopenia as a significant prognostic factor in metastatic RCC.^10^

Ueki et al. identified PMI as a significant prognostic factor for overall survival in patients with RCC receiving nivolumab therapy.^39^

The results of the present study regarding the potential usefulness of preoperative PMI measurement to predict clinical outcomes in RCC patients appear in line with these previous studies by demonstrating an association between PMI as an indicator of sarcopenia and survival in patients with RCC. A new observation, that was not reported previously for RCC, is that this association between CT-based measurements of sarcopenia may be stronger in men compared with women. For gastric cancer patients, a recent study by Lee et al. found that postoperative loss of muscle mass and sarcopenia were significant prognostic factors for survival only in men.^38^

Sex-specific differences in skeletal muscle are assumed to result from different hormone levels such as testosterone and estrogen, as well as differences in gene expression.^40^ A previous study showed that older men experienced a higher leg skeletal muscle (LSM) loss compared with women during a follow-up time of several years.^41^ Assuming that age-related loss of muscle mass is greater and often happens more quickly in men than in women, the impact on mortality in male RCC patients may be more severe in men than in women.

To our knowledge, the present study is the first approach in RCC patients to simultaneously examine tumor characteristics and CT-quantified body composition (abdominal fat, psoas muscle density), taking into account sex differences. Our study design has several advantages. First, we took a holistic approach to evaluate sex-specific differences in RCC, considering not only abdominal adipose tissue distribution and psoas muscle density but also patient age, tumor size, and pathology. Second, two experienced board-certified radiologists measured/labeled the areas of abdominal adipose fat tissue and psoas muscle, which is a reliable method to determine and quantify the tissue areas. Third, we used a rigorous statistical methodology to determine the overall as well as sex-specific effects of abdominal adipose tissue distribution and sarcopenia on RCC at diagnosis.

Limitations of our study include the relatively small number of patients and the retrospective, non-randomized single-center design with a consequent probability of selection bias. In addition, survival data were not available for all patients. Another limitation might be that obese or sarcopenic patients with aggressive RCC may sometimes be considered unsuitable for surgery and therefore not be analyzed in our present study, which focused on patients receiving surgical treatment. Finally, further studies are required to validate our findings in a different data set and determine optimal cut-off values for the PMI.

A higher PMI was independently associated with longer overall survival in RCC patients undergoing (partial) nephrectomy, with a stronger association in men compared with women. In addition, the PMI showed an inverse association with tumor grade, whereby this association was slightly more pronounced in women than in men.

CT-based measurement of the psoas muscle area may therefore be convenient and easy to acquire for estimating individual prognosis, especially in men.
